# Self-propelled ion gel at air-water interface

**DOI:** 10.1038/s41598-017-09351-6

**Published:** 2017-08-24

**Authors:** Kazuaki Furukawa, Tetsuhiko Teshima, Yuko Ueno

**Affiliations:** 1 School of Science and Engineering, Meisei University, Hino Tokyo, 191-8506 Japan; 2 NTT Basic Research Laboratories, NTT Corporation, Atsugi, Kanagawa 243-0198 Japan

## Abstract

We report on a self-propelled gel using ionic liquid as a new type of self-propellant that generates a powerful and durable motion at an air-water interface. The gel is composed of 1-ethyl-3-methylimidazolium-bis(trifluoromethylsulfonyl)imide (EMIM-TFSI) and poly(vinylidene fluoride-co-hexafluoropropylene) (P(VDF-co-HFP)). A long rectangular ion gel piece placed on the interface shows rapid rotation motion with maximum frequency close to 10 Hz, corresponding to the velocity over 300 mms^−1^ at an outmost end of the piece. The rotation continues for ca. 10^2^ s, followed by a reciprocating motion (<~10^3^ s) and a nonlinear motion in long-time observations (>~10^3^ s). The behaviours can be explained by the model considering elution of EMIM-TFSI to the air-water interface, rapid dissolution into water, and slow diffusion in an inhomogeneous polymer gel network. Because the self-propellants are promptly removed from the interface by dissolution, durable self-propelled motions are observed also at limited interface areas close in size to the gel pieces. A variety of motions are induced in such systems where the degree of freedom in motion is limited. As the ion gel possesses formability and processability, it is also advantageous for practical applications. We demonstrate that the gel does work as an engine.

## Introduction

Synthetic materials that show spontaneous motions similar to life draw much attention. In order to drive the self-propelled motions, sophisticated mechanisms are built in to these materials. They are recently called active matter^1–3^, distinguished from the ones in which the motions are driven by the external power sources^4–18^. The active matter includes biological materials and systems such as molecular motors and cells as well, and is discussed in physics in terms of non-equilibrium systems. In most of the cases, the self-propelled motion relies on the conversion from chemical energy to mechanical energy called chemomechanical conversion. This is almost an isothermal process, which is very different from thermal engine that creates most of the energy consumed in our daily life^4^. There are a few kinds of non-biological materials that show self-propelled characteristics thus far reported. One example is an oil droplet in water or a water droplet in oil^19–22^. Droplets are flexible and often accompany deformation of the shape showing ameba-like motion^23^. Another is a catalytic nanoparticle that generates propulsion by electrochemically decomposing water^24–26^.

Polymer gel is a more promising material because it is a scalable solid and maintains formability and processability. They can be advantages and great potential for the polymer gel in practical applications. An example is reported that includes chemical reagents to initiate Belousov-Zhabotinsky (BZ) reaction^27–29^. Periodic change in volume of the gel induced by the periodic chemical BZ reaction drives a self-propelled motion. It is also reported a polymer gel that releases embedded moieties inside the gel for driving motion^30–32^. The direction of the motion can be controlled by a shape of the gel, taking advantage of its processability^32^. The self-propelled motions are, however, rather inferior in power and durability compared with those realized by the system using external power supply. A hint to overcome the drawbacks is to design a new self-propellant. For instance, a material using peptide as a self-propellant was recently reported, in which the released peptides form a self-assemble structure including a hydrophobic part that contributes to enhanced propulsion^33^.

Our strategy to achieve more powerful and durable self-propelled motion in a gel system is to choose an ionic liquid as a new self-propellant. The ionic liquid is a 1:1 mixture of organic cation and anion, and is a stable material with high heat resistivity and with low chemical activity and sublimability. We show that a polymer gel containing an ionic liquid, called an ion gel^34^, exhibits powerful and durable self-propelled motions at an air-water interface. The motions occur at the interface between air and pure water, and common aqueous solutions of NaCl and phosphate as well. The motions also occur at limited interface areas close in size to the gel piece, which is unique for our gel. The results are rationalized by the following mechanism: the motions are driven by the elution of the ionic liquid from the gel to the interface, and the ionic liquid at the interface is promptly removed by being dissolved in water. The inhomogeneous gel structure and the instability of the system makes the gel motions non-equilibrium and nonlinear. This is encouraged by the inhomogeneous diffusion of the ionic liquid inside the gel. We also demonstrate that the gel does work as an engine at an air-water interface. We should note that the ion gel has been extensively used for applying gate voltage to electronic devices^34–38^. Despite of this, its characteristics in the presence of water have not been positively investigated. This is because water causes noise during electric measurements due to its electrochemical decomposition and is thoroughly eliminated from the ion gel in the previous studies. Our present study reveals a hidden unknown property of ion gels: a significant self-propelled characteristic at the air-water interface. This can offer the ion gels an entirely new role that is useful for not only fundamental research concerning to non-equilibrium systems and active matters but also applications including micro motors, microfluidics and soft actuators.

## Results and Discussion

**Figure 1:**
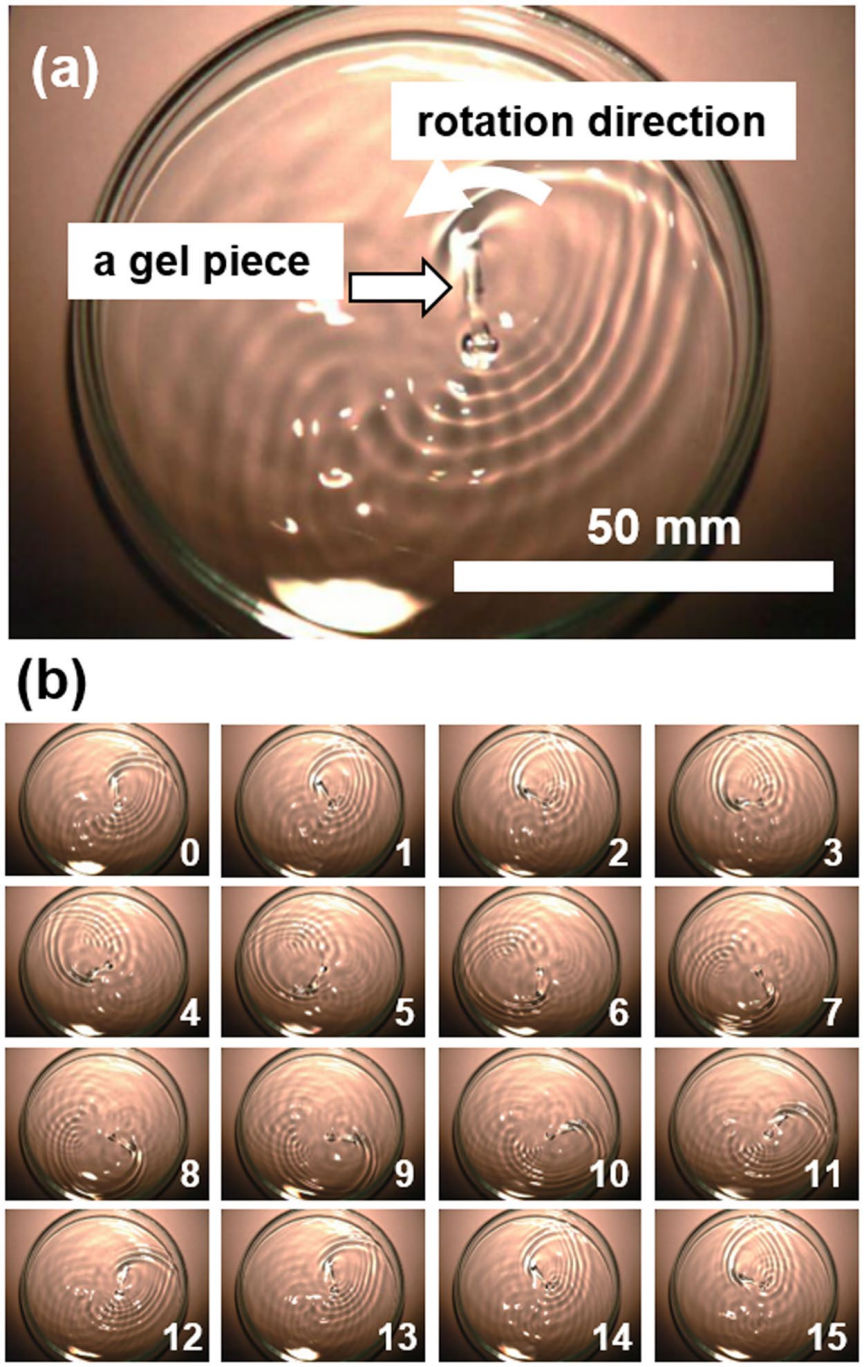
(**a**) A long rectangular gel piece (approximately 2 × 15 × 0.5 mm) placed on a distilled water surface in a petri dish with a diameter of 90 mm. Scale bar: 50 mm. (**b**) Time lapse images of the gel piece. Interval: 0.01 s.

Figure 1 shows a typical ion gel motion at an air-water interface. The shape of the gel piece is a long rectangle with dimensions approximately 2 (width) × 15 (length) × 0.5 (thickness) mm (Fig. 1, Supplementary Movie S1). The gel piece starts a rotation motion when it is placed on distilled water in a 90 mm- diameter petri dish. The rotation motion has been recorded by high speed camera with 1000 frames per second. The images with 0.01 s interval are shown in Fig. 1b. After 13 images, the gel moves back to the same position, which means the gel piece rotates within 0.13 s or with ca. 8 Hz.

**Figure 2:**
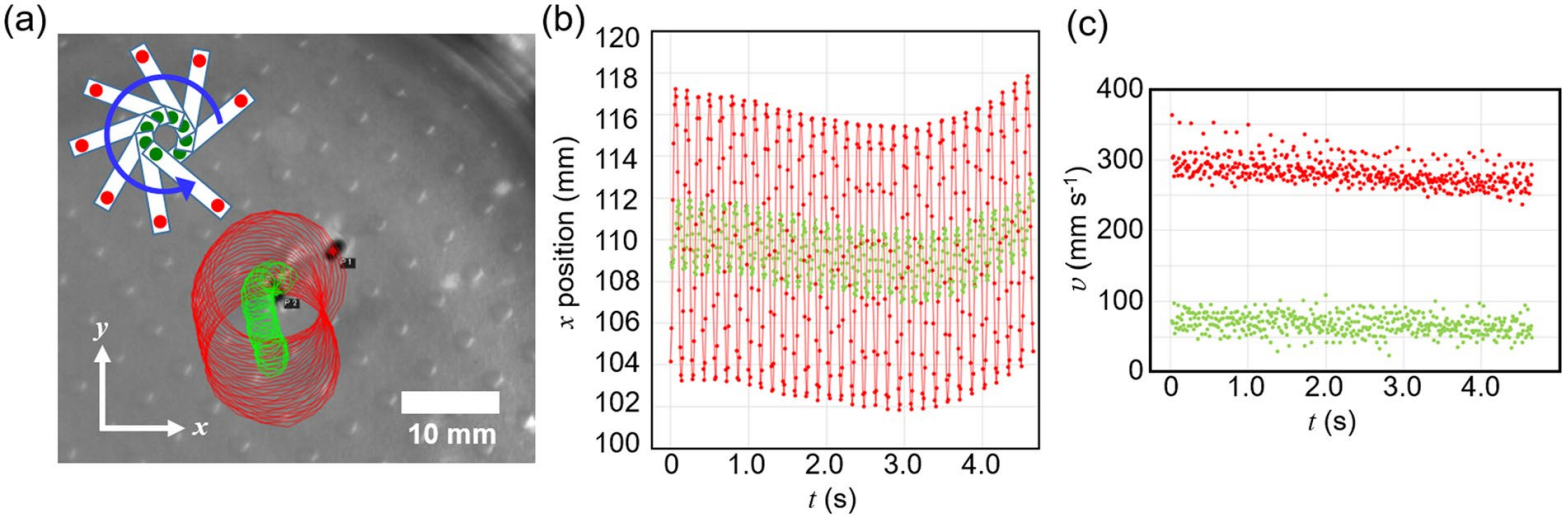
(**a**) Traces of two marks (indicated by red and green) put on a gel piece within a couple of seconds with 100 frames per second. The insertion sketch schematically shows the rotation direction of the piece with the mark positions in red and green. (**b**) Plot of *x* position over time, *t*. (**c**) Plot of velocity, *v*, over *t*.

We put two marks close to the both ends of another long gel piece for tracking and analysing the motion. Traces of two marks of the gel piece within a couple of seconds are shown in Fig. 2a. Both traces draw continuous circles with different radii. They also show a gradual drift of the centre of the circles. The *x*-axis position of the marks over time, *t*, is plotted in Fig. 2b. The sign curve oscillation of *x*-axis position becomes clear, which corresponds to the nearly constant-speed rotation motion. The oscillation amplitude of one mark is about 14 mm, while that of the other 3.5 mm. This indicates that the rotation centre does not match the centre of the rectangle. The gradual *x*-position peak shift corresponds to the shift of the centre of the gel rotation. The velocity, *v*, over *t* is plotted in Fig. 2c. It reaches 300 mms^−1^ at the mark position. Because the marks are not exactly at the ends of the long sides, instead at ca. 1 mm inner position from the both ends, the velocity at the outmost end can reach more than the value observed in Fig. 2c. The velocity is larger than the reported values for self-propelled materials. For instance, 40 mms^−1^ has been reported for a droplet on glass surface placed in liquid^19^, and 67 mms^−1^ for a metal-organic framework material^33^.

The ion gel piece does not show any kinetic motion when it is fully immersed in water. This means that the specific gravity of the gel is greater than that of water. If a part of the gel is exposed at an air-water interface, it shows kinetic motion. For instance, if we expose only one end of a long gel piece at an air-water interface, the gel shows the precession motion as the other end inside water become a cone tip facing downward. In addition, we confirm that gel motion is observed with aqueous 100 mM NaCl and 100 mM phosphate buffer. However, the gel motion is immediately stopped when we add a small amount of surfactant on the water surface.

**Figure 3:**
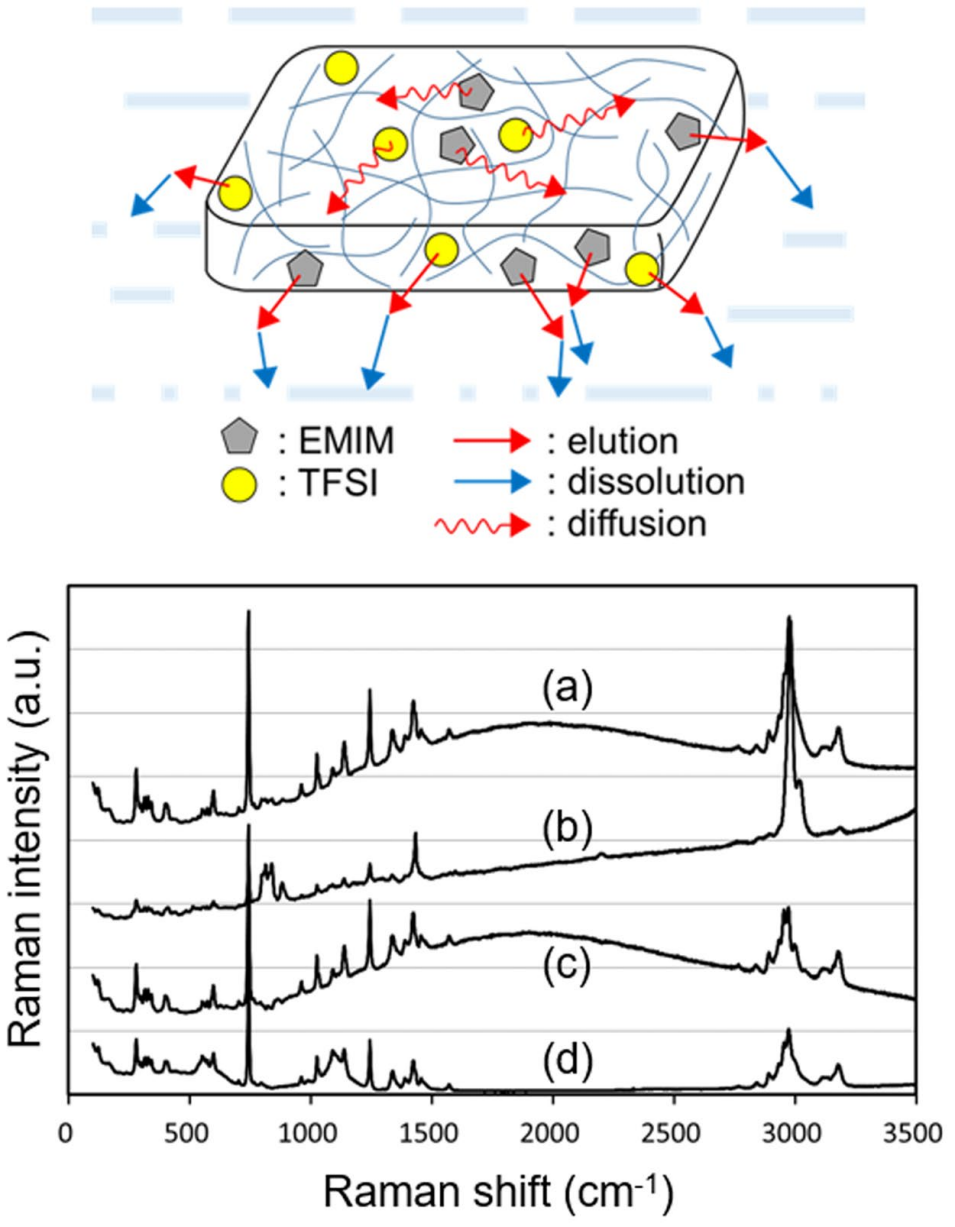
Top: schematic illustration of the mechanism of self-propelled motion of the ion gel. Bottom: Raman spectra of (**a**) an original ion gel; (**b**) an ion gel after kinetic motion; (**c**) subtraction of (**a**) and (**b**); (**d**) EMIM-TFSI. The vertical positions of the spectra are biased for clarity.

These results indicate that the air-water interface is an important field for generating the propulsion for driving the ion gel motion. The ion gel is composed of only two materials, a polymer P(VDF-co-HFP) and an ionic liquid EMIM-TFSI, both of which are stable and inert with water. We can thus propose the mechanism for the self-propelled motion as illustrated in Fig. 3. In the model, EMIM and TFSI eluted from the gel to the air-water interface generate the surface tension gradient that is large enough for driving the powerful and durable rotation motions. The mechanism is basically the same with known Marangoni effect for driving “camphor boat”^39, 40^. The difference in mechanism between the ion gel and the camphor boat appears in the next important step, how the molecules that generate the surface tension gradient are removed from the interface for the durable motion. In the camphor boat system, camphor is readily sublimed, which is advantageous for its continuous motion. This principle is also applied to the self-propelled gel containing ethanol as a propellant^32^. By contrast, EMIM-TFSI does not sublime. We propose the model that EMIM and TFSI at the interface are promptly removed by being dissolved into water. The solubility of P(VDF-co-HFP) in water is negligible, but that of EMIM-TFSI is determined to be 4.1 × 10^−2^ mol·dm^−3^ (1.6 × 10^1^ g·dm^−3^).

If the elution of self-propellant is homogeneous, the sum of the generated force must be balanced. In spite of this, the gel piece exhibits powerful and durable motion. This is due to several reasons that break the balance. First, the shape of the gel is not perfectly symmetric. Thus the elution of EMIM-TFSI becomes anisotropic. The second is the inconstant elution of EMIM-TFSI. This is caused by a structural inhomogeneity of the gel in a microscale, which an amorphous material like gel intrinsically possesses. In addition to these material-derived effects, the other possibility is due to a spontaneous breaking of the symmetry. Once a gel piece starts kinetic motion by perturbations or fluctuations, it induces an asymmetrical profile of EMIM-TFSI concentration in the surrounding interface area. This effect has explained the motion of camphor piece driven by Malangoni effect^39^ and self-propelled particles^33^. Although quantitative estimation of each effect on the motion is difficult, we can claim that EMIM-TFSI works as a powerful self-propellant in the ion gel. We can also claim that the diffusion rate is rather slow, judging from the duration of the motion. The rate is most likely affected by the interaction between the ionic liquid and the amorphous gel network, as well as the hydrophobic characteristic of P(VDF-co-HFP). The present gel realizes a good combination of two materials. There has been reported an example to use an ionic liquid as a self-propellant^41^. The velocity of the moving ionic liquid droplet in the example is much slower than ours. The motion of the droplet is, however, driven by the Cl^−^ concentration gradient prepared in a microchannel, hence the mechanism is different from our present case.

In order to clarify the elution of EMIM-TFSI from the ion gel during the motion, we observe Raman spectra of the gel before (Fig. 3a) and after (Fig. 3b) kinetic motion. The subtraction spectrum (Fig. 3c) agrees well with the spectrum observed for EMIM-TFSI itself (Fig. 3d), which confirms the elution of both EMIM and TFSI. We also examined that the gel piece does not show motion on the surface of saturated EMIM-TFSI aqueous solution (Supplementary Movie S2). Since the saturated solution cannot dissolve EMIM-TFSI, the gel piece could not obtain the kinetic force in the conditions.

To further check the validity of the proposed model, we examine if the gel shows the durable motion at limited air-water interface areas that are close in size with the ion gel pieces. Because EMIM and TFSI is removed promptly from the interface by being dissolved into water in our model, the duration of the motions must be maintained. This also corresponds to observing the ion gel motion in the limited degree of freedom.

**Figure 4:**
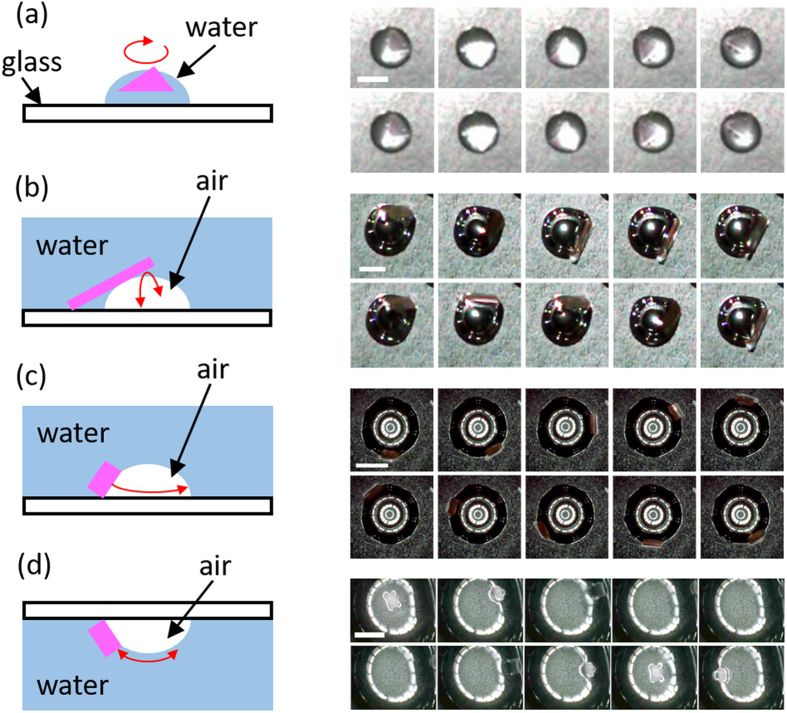
Left: Schematic drawings of the experimental conditions. (**a**) A triangle gel piece on a water droplet. (**b**) A long rectangular gel piece at a bubble surface in water. (**c**) A tetragonal gel piece at a bubble surface in water. (**d**) A tetragonal gel piece at a bubble surface beneath a glass substrate in water. The red arrows indicate the direction of motion of the gel pieces. Right: Frame-by-frame images observed for each conditions. Scale bar and interval time are (**a**) 5 mm, 0.050 s, (**b**) 2 mm, 0.033 s, (**c**) 5 mm, 0.064 s, and (**d**) 5 mm, 0.040 s. See also Supplementary Movies S3–S6.

Our scheme is shown in Fig. 4. We prepare the limited interface areas by a water droplet in ambient conditions or a bubble in water. We also change the shapes of the gel which are schematically shown in Fig. 4. In every case, the gel shows kinetic motions at the air-water interfaces. Figure 4a shows a rotation motion when a triangle gel piece is put on top of a water droplet. In this case the diameter of the droplet and the edge length of the triangle gel are comparable, and the gel shows the rotation motion (Supplementary Movie S3). Figure 4b and c are the cases when we use the air-water interface prepared using bubbles in water. We put a long rectangular gel piece so that the one end is touched on the glass substrate, thus the piece bridges the glass and the air-water interface (Fig. 4b, Supplementary Movie S4). We observe a reciprocating motion as the end of the gel piece on the glass becomes a pivot point. We also put a tetragonal piece whose edges are shorter than the circumference of the bubble (Fig. 4c, Supplementary Movie S5). We observe a rotation motion of the gel piece along the circle edge made by the bubble in contact with the glass substrate. In this case, the tetragonal gel piece also rotates by itself and moves along the circle edge. It should be noted that we observe the reverse of the rotation direction in a long-time observations (Supplementary Movie S5). We further observe a motion of a tetragonal gel piece at the interface prepared by a bubble beneath a glass substrate in water (Fig. 4d, Supplementary Movie S6). The gel piece shows a reciprocating motion in this case, which has never been observed under the conditions in Fig. 4c. The elution of EMIM-TFSI also appears as a change in the gel piece shape during the motion in Fig. 4c (Supplementary Movie S5.). The gel was slightly warped so that the gel shape becomes an arch-like shape convex to the air at the air-water interface. This is because the gel volume becomes smaller on the side facing to water where the gel is shrunk by the EMIM-TFSI elution.

It has been reported that the self-propelled motions can be controlled by regulating the mobile areas. For instance, a camphor grain in a linear water channel showed oscillation motion along the channel^42^, and two camphor boats in triangle or square-shape water channel showed synchronized motions^43^. The essential difference between the camphor systems and ours is, as we discussed above, the reduction mechanism of the molecules at air-water interfaces. Without this mechanism, the self-propelled motions at the air-water interface close in size to the gel piece, as shown in Fig. 4, have never been driven.

**Figure 5:**
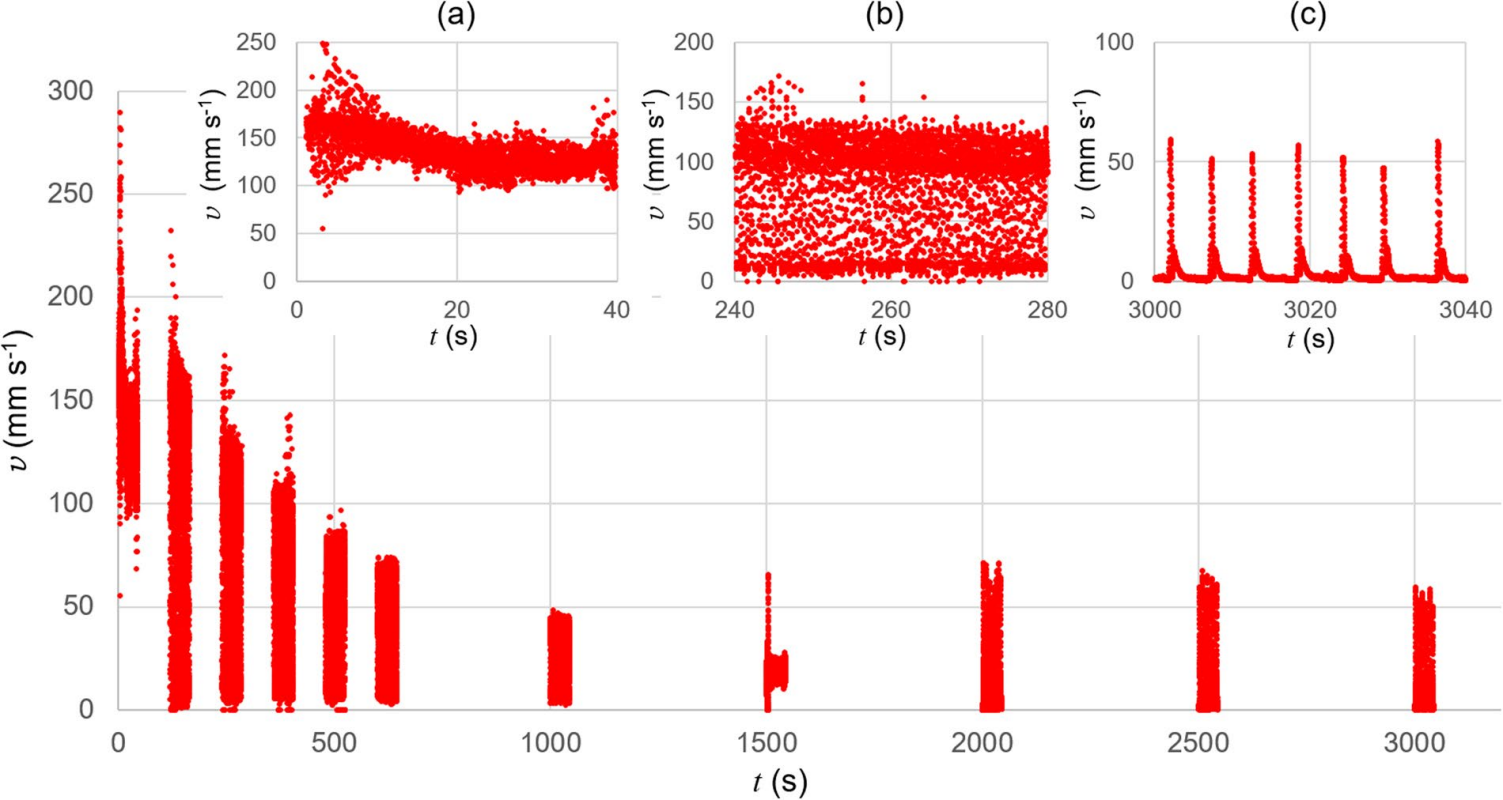
A plot of velocity, *v*, of a mark put on a rectangular gel piece over time, *t*. The motion is recorded with 100 frames per second for ca. 45 s with an interval of 120 s for the first 6 observations (0 ≤ *t* ≤ ~645) and of 500 s for the rest (*t* ≥ 1000). The insertions are the magnifications of the plot at (**a**) 0 ≤ *t* ≤ 40, (**b**) 240 ≤ *t* ≤ 280, (**c**) 3000 ≤ *t* ≤ 3040.

We execute long-time observations of the ion gel motion to confirm the duration of the motion. Figure 5 plots *v* over a wide range of *t* for a long rectangular ion gel. Because of the limitation of high speed camera system, we can record ca. 45 s for a single experiment. Thus the gel motion images are recorded in the time interval indicated in the legend in Fig. 5. The longer *t* is, the smaller *v*, because the gel piece is losing EMIM-TFSI, the self-propellant for the motion. The decrease of *v* is exponential, but it looks that *v* is not reaching 0 even after several 10^3^ s. The gel piece shows different kinetic behaviours depending on the time range, as shown in the insertions in Fig. 5. The motion of the gel piece is rotational in the early stage, in which *v* is slowly decreasing (Fig. 5a, Supplementary Movie S7). Following this stage, the gel starts to show a reciprocating motion where the piece in a translation motion is periodically bounced at the wall of a petri dish (Fig. 5b, Supplementary Movie S8). This second stage continues until *t* ~ 10^3^ s. While it is not easily seen in the plot in Fig. 5, the gel motion is almost stopped at *t* over several 10^3^ s. In this third stage, however, the gel piece shows sudden displacement non-periodically. It appears as the occasional observations of finite value of *v* in Fig. 5c, where *v* = 0 at most of the time between 3000 ≤ *t* ≤ 3040 (Supplementary Movie S9). This nonlinear kinetic behaviour is one of the characteristic features of our gel, which can be firmly explained by our model (Fig. 3). The fast motions persisting for the first and second stages are due to the continuous elution of EMIM-TFSI until EMIM-TFSI near the gel surface is exhausted. However, in the central part of the gel is contained more self-propellants that have to travel through the inhomogeneous gel network to reach close to the gel surface. The time required is long and inconstant, and the propulsion by a small amount of EMIM-TFSI is great enough for driving the motion. This process is in consistent with the slow release of the self-propellant, thus the durable motion of the ion gel piece.

Finally, we address possibility of the practical use of our ion gel. As the ion gel consists of non-sublimable materials, we can load it into a vacuum chamber for depositing a poly(chloro-*p*-xylylene) (parylene-C) thin film. The parylene-C-coated gel piece does not move on an air-water interface because EMIM-TFSI cannot pass through a conformal parylene-C coating. If we cut a part of the parylene-C, the gel piece resumes a self-propelled motion by the elution of EMIM-TFSI from the exposed area (Supporting information, Supplementary Movie S10). This can be an advantage for the ion gel to drive the motion in a controllable manner. The motion is observed even after 24 h in this case, because the elution of EMIM and TFSI is allowed only from the cutting section. We also demonstrate the use of the gel as an engine to drive the artificial structure at an air-water interface. A small gel piece, approximately 4 mm square in size, can rotate the large structure made by plastic with dimensions of 80 × 16 × 0.5 mm (Supporting information, Supplementary Movie S11). The rotation direction is clockwise if we design the structure in Fig. S1. This includes a technical merit that the direction of motion can be controlled by combining the ion gel with other structural materials. The design is also advantageous to regulate the direction of EMIM-TFSA elution, which can more efficiently convert the chemical energy to the kinetic energy.

## Conclusions

In conclusion, we report for the first time the potential of an ion gel as a new type of self-propelled materials at air-water interface. The ion gel, composed of EMIM-TFSI and P(VDF-co-HFP) in this study, shows powerful and durable self-propelled motions with the maximum velocity over 300 mms^−1^ with the duration time ~10^2^ s. The duration time is even longer, over 10^3^ s, as the motion continues by changing its mode: from rotation to reciprocating, and finally nonlinear motion. The mechanism is explained by Marangoni effect. This is commonly known for driving a camphor boat, but the difference appears in the reduction mechanism of the self-propellant molecules at the interface. We propose a new model in which the ionic liquid is promptly reduced from the interface by being dissolved into water for the durable motion. This model is supported by our new observations of durable motions of the ion gel at the confined air-water interfaces. The observations also reveal that a variety of motions can be induced by designing the sizes and shapes of the interface and the ion gel piece. Our finding is also valuable for practical applications using the ion gel as an engine. We demonstrate two examples: an inert parylene film-coated ion gel that can start the self-propelled motion by breaking the coating and a combined structure with a plastic material that generates the regulated motion. This is the first report of an ion gel to be used as a self-propelled material. A variety of ionic gels will be examined using different ionic liquids, as well as different polymers, for developing those with more powerful and durable motions. The ion gel shows the nonlinear behaviour, which must be of interest also as a new type of active matter.

## Methods

1-Ethyl-3-methylimidazolium-bis(trifluoromethylsulfonyl)imide (EMIM-TFSI) was purchased from Iolitech, poly(vinylidene fluoride-co-hexafluoropropylene) (P(VDF-co-HFP)) was from Aldrich, acetone from Kanto chemical co., Inc. The ion gel was prepared according to the previous report^36^. In detail, a solution of P(VDF-co-HFP) in acetone (1:7 in weight) was further mixed with EMIM-TFSI. The mixture ratio is fixed as solution:EMIM-TFSI = 2:1 in weight for the ion gel shown in this study. The mixed solution (ca. 2 g) is poured into a glass vial with a flat bottom (an inner diameter of ca. 36 mm) with a screw cap. The gradual vaporization of acetone from the mixture, typically a couple of days by loosening the screw cap at room temperature, forms a transparent ion gel film. The film was then dried under vacuum for overnight to remove remaining acetone. The obtained ion gel film was cut into a small piece of required size and shape by a knife.

For observing the typical motions of a long rectangular gel piece at large air-water interface, we gently put the piece on the surface of DI water half-filled of a 90 mm-diameter petri dish. Two marks close to the both ends were put prior to the observations when tracking and analysing the motions. On the other hand, the confined air-water interfaces, which are schematically shown in Fig. 4, were prepared by a drop of DI water on a cover slip, by a bubble on a cover slip sunk in DI water, or by a bubble beneath a cover slip immersed in DI water. Depending on the purpose and the confined area, the ion gel film was cut into a triangle, rectangular or tetragonal piece. In these experiments, we added a small amount of ethanol solution of rhodamine in the mix solution before acetone vaporization in the gel film preparation. This made a gel coloured and resulted in better visualizations.

Motions of the gel piece was recorded by high speed camera systems (HAS-L2, Ditect co. ltd, Japan and VW-9000, Keyence, Japan) and analysed by their own software (DIPP-Motion V/2D and VW-9000 MotionAnalyzer).

## Electronic supplementary material

Supplementary information

Supplementary Movie S1

Supplementary Movie S2

Supplementary Movie S3

Supplementary Movie S4

Supplementary Movie S5

Supplementary Movie S6

Supplementary Movie S7

Supplementary Movie S8

Supplementary Movie S9

Supplementary Movie S10

Supplementary Movie S11
