# Supplementary material for: Self-propelled ion gel at air-water interface
Source: Sci Rep. 2017 Aug 24;7:9323. doi: 10.1038/s41598-017-09351-6 (PMC5571170; doi:10.1038/s41598-017-09351-6)
Supplement: Supplementary file 1 — Supplementary information [file 41598_2017_9351_MOESM1_ESM.pdf]

Supplementary information of "Self-propelled ion gel at air-water interface"

By Kazuaki Furukawa,[1,*] Tetsuhiko Teshima,[2] Yuko Ueno[2]

[1]Meisei University, Hino Tokyo 191-8506 Japan

[2]NTT Basic Research Laboratories, NTT Corporation, Atsugi Kanagawa 243-0198 Japan

Email: kazuaki.furukawa@meisei-u.ac.jp

Coating of ion gel by poly(chloro-*p*-xylylene)

A layer of poly(chloro-*p*-xylylene) (parylene-C) 1 μm in thick was deposited using a chemical deposition system (LABCOTER PDS2010, USA). Dichloro-di(*p*-xylylene) (DPX-C, USA) powder was purchased from Speedline Technology, USA. Inside the deposition system, the source powder was first vaporized at 150°C, and then pyrolyzed at 690°C to generate chloro-*p*-xylylene monomer. A reduction in the chamber temperature caused chloro-*p*-xylylene to condense onto the surface of ion gel pieces to form conformal parylene-C membrane coating. The thickness was determined by the weight of the initially loaded dichloro-di(*p*-xylylene) using a rate of approximately 6.25×10^{-1} μm /g.

Fabrication of a plastic structure

A structure that is driven by a small gel piece in Supplementary Movie S11, was fabricated by 3D printer Objet24 (Stratasys, Eden Prairie, MN, USA) using VeroWhite FullCure835. The shape and dimensions are in Fig. S1.

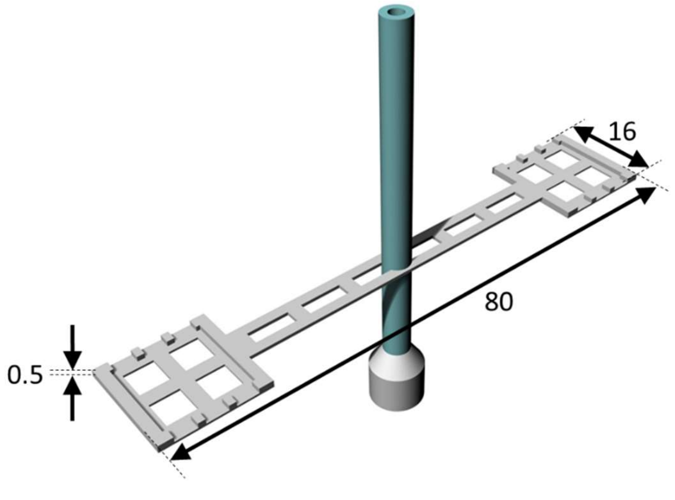

Figure S1. Dimensions and shape of a structure used in Movie S11. Dimensions are in mm.

Supplementary Movie list

Supplementary Movie S1.

A slow motion movie (×0.015) for a typical rotation motion of a long rectangle ion gel piece at air-water interface.

Supplementary Movie S2.

A real time movie for motions of a long rectangle ion gel piece at the interface between air and water (left), aqueous saturated solution of EMIM-TFSI (right), and again water (left).

Supplementary Movie S3.

A slow motion movie (×0.25) for a rotation motion of a triangle ion gel piece at a water droplet surface.

Supplementary Movie S4.

A slow motion movie (×0.25) for a reciprocating motion of a long rectangle gel piece at a surface of a bubble in water.

Supplementary Movie S5.

A slow motion movie (×0.12) for a rotation motion of a square gel piece at a surface of a bubble in water.

Supplementary Movie S6.

A slow motion movie (×0.2) for a reciprocating motion of a square gel piece at a surface of a bubble in water formed beneath a glass substrate.

Supplementary Movie S7.

A slow motion movie (×0.3) for a rotation motion of a long rectangle gel piece in the early stage ($0 < t < \sim 10^2$, Fig. 6a) after the piece is placed on the water surface.

Supplementary Movie S8.

A slow motion movie (×0.3) for a motion of the long rectangle gel piece in Supplementary Movie S7 in the middle stage ($\sim 10^2 < t < \sim 10^3$, Fig. 6b) after the piece is placed on the water surface.

Supplementary Movie S9.

A slow motion movie (×0.3) for a motion of the long rectangle gel piece in Supplementary Movie S7 in the late stage ($t > \sim 10^3$, Fig. 6c) after the piece is placed on the water surface.

Supplementary Movie S10.

A real time movie for motions of a long rectangle gel piece coated by 1 μm thick poly(chloro-*p*-xylylene) at air-water interface before and after cutting at the center position of the gel by a knife.

Supplementary Movie S11.

A real time movie for a controlled rotation motion of a plastic structure in Fig. S1 using a small gel piece placed at one corner of the structure.
